# Prevalence, risk factors, and uptake of interventions for sexually transmitted infections in Britain: findings from the National Surveys of Sexual Attitudes and Lifestyles (Natsal)

**DOI:** 10.1016/S0140-6736(13)61947-9

**Published:** 2013-11-30

**Authors:** Pam Sonnenberg, Soazig Clifton, Simon Beddows, Nigel Field, Kate Soldan, Clare Tanton, Catherine H Mercer, Filomeno Coelho da Silva, Sarah Alexander, Andrew J Copas, Andrew Phelps, Bob Erens, Philip Prah, Wendy Macdowall, Kaye Wellings, Catherine A Ison, Anne M Johnson

**Affiliations:** a Research Department of Infection and Population Health, University College London, Mortimer Market Centre, London, UK; b NatCen Social Research, London, UK; c Virus Reference Department, Public Health England, London, UK; d Centre for Infectious Disease Surveillance and Control, Public Health England, London, UK; e Sexually Transmitted Bacteria Reference Unit, Public Health England, London, UK; f Department of Health Services Research and Policy, London School of Hygiene and Tropical Medicine, London, UK; g Department of Social and Environmental Research, London School of Hygiene and Tropical Medicine, London, UK

## Abstract

**Background:**

Population-based estimates of prevalence, risk distribution, and intervention uptake inform delivery of control programmes for sexually transmitted infections (STIs). We undertook the third National Survey of Sexual Attitudes and Lifestyles (Natsal-3) after implementation of national sexual health strategies, and describe the epidemiology of four STIs in Britain (England, Scotland, and Wales) and the uptake of interventions.

**Methods:**

Between Sept 6, 2010 and Aug 31, 2012, we did a probability sample survey of 15 162 women and men aged 16–74 years in Britain. Participants were interviewed with computer-assisted face-to-face and self-completion questionnaires. Urine from a sample of participants aged 16–44 years who reported at least one sexual partner over the lifetime was tested for the presence of *Chlamydia trachomatis*, type-specific human papillomavirus (HPV), *Neisseria gonorrhoeae*, and HIV antibody. We describe age-specific and sex-specific prevalences of infection and intervention uptake, in relation to demographic and behavioural factors, and explore changes since Natsal-1 (1990–91) and Natsal-2 (1999–2001).

**Findings:**

Of 8047 eligible participants invited to provide a urine sample, 4828 (60%) agreed. We excluded 278 samples, leaving 4550 (94%) participants with STI test results. Chlamydia prevalence was 1·5% (95% CI 1·1–2·0) in women and 1·1% (0·7–1·6) in men. Prevalences in individuals aged 16–24 years were 3·1% (2·2–4·3) in women and 2·3% (1·5–3·4) in men. Area-level deprivation and higher numbers of partners, especially without use of condoms, were risk factors. However, 60·4% (45·5–73·7) of chlamydia in women and 43·3% (25·9–62·5) in men was in individuals who had had one partner in the past year. Among sexually active 16–24-year-olds, 54·2% (51·4–56·9) of women and 34·6% (31·8–37·4) of men reported testing for chlamydia in the past year, with testing higher in those with more partners. High-risk HPV was detected in 15·9% (14·4–17·5) of women, similar to in Natsal-2. Coverage of HPV catch-up vaccination was 61·5% (58·2–64·7). Prevalence of HPV types 16 and 18 in women aged 18–20 years was lower in Natsal-3 than Natsal-2 (5·8% [3·9–8·6] *vs* 11·3% [6·8–18·2]; age-adjusted odds ratio 0·44 [0·21–0·94]). Gonorrhoea (<0·1% prevalence in women and men) and HIV (0·1% prevalence in women and 0·2% in men) were uncommon and restricted to participants with recognised high-risk factors. Since Natsal-2, substantial increases were noted in attendance at sexual health clinics (from 6·7% to 21·4% in women and from 7·7% to 19·6% in men) and HIV testing (from 8·7% to 27·6% in women and from 9·2% to 16·9% in men) in the past 5 years.

**Interpretation:**

STIs were distributed heterogeneously, requiring general and infection-specific interventions. Increases in testing and attendance at sexual health clinics, especially in people at highest risk, are encouraging. However, STIs persist both in individuals accessing and those not accessing services. Our findings provide empirical evidence to inform future sexual health interventions and services.

**Funding:**

Grants from the UK Medical Research Council and the Wellcome Trust, with support from the Economic and Social Research Council and the Department of Health.

## Introduction

Diagnosed sexually transmitted infections (STIs) have increased substantially in Britain (England, Scotland, and Wales) since the 1990s^1^ and throughout the past decade to the end of 2012,^2^ emphasising the importance of sustained public health programmes to identify and treat infections, reduce morbidity and mortality, and prevent onward transmission. Population-based estimates of infection prevalence, risk distribution, and intervention uptake inform the design and delivery of STI control programmes. The National Surveys of Sexual Attitudes and Lifestyles (Natsal)-1 (1990–91) and Natsal-2 (1999–2001) have guided the development of sexual health programmes by providing empirical evidence to understand the heterogeneity of sexual behaviour in the British population,^3^, ^4^ the prevalence of STIs, and the drivers of transmission.^5^, ^6^ Surveillance of STI diagnoses does not measure the true prevalence of STIs in the population because infections are often asymptomatic and undiagnosed. Natsal links prevalence (measured with biological sampling) with population risk factors and can thus assess the extent to which infected and at-risk individuals access specific interventions and services.

Since Natsal-2, several strategies to improve sexual health have been implemented in Britain, as they have been in other developed countries or regions.^7^, ^8^, ^9^, ^10^, ^11^, ^12^ The British initiatives include the National Strategy for Sexual Health and HIV in England (2001);^13^ Respect and Responsibility: Strategy and Action Plan for Improving Sexual Health in Scotland (2005);^14^ and the Strategic Framework for Promoting Sexual Health in Wales (2001).^15^ All the strategies have broad sexual health objectives and promote a reduction in risk behaviour (eg, increased condom use). Three STI-specific interventions have been implemented: (1) the English National Chlamydia Screening Programme (NCSP) in 2003 (opportunistic screening in sexually active women and men aged 16–24 years); Scottish and Welsh strategies encouraged testing in this age group, but with no formal programme; (2) the UK human papillomavirus (HPV) immunisation programme in 2008, using the bivalent HPV 16/18 vaccine (routine immunisation of girls aged 12–13 years, with a catch-up programme up to age 17 years); and (3) strategies to increase HIV testing in target groups as outlined in the 2008 British HIV Association (BHIVA) National Guidelines.^16^ Furthermore, the range and accessibility of STI services has broadened, with modernised sexual health (genitourinary medicine [GUM]) clinics, targets for 48 h waiting times, and expansion of the role of primary care.

We present age-specific and sex-specific estimates for the population prevalence of, and associated risk factors for, infection with *Chlamydia trachomatis*, high-risk HPV, *Neisseria gonorrhoeae*, and HIV from a probability sample of 16–44-year-olds in Britain. Additionally, we describe the uptake of interventions and sexual health services, and explore changes since Natsal-1 and Natsal-2.

## Methods

### Participants and procedures

Natsal-3 is a stratified probability sample survey of 15 162 women and men aged 16–74 years in Britain who were interviewed between Sept 6, 2010, and Aug 31, 2012. The estimated overall response rate was 57·7% and the cooperation rate was 65·8% (of all eligible addresses contacted). Participants were interviewed with a combination of computer-assisted face-to-face and self-completion questionnaires, which included questions about participants' sexual lifestyles and attitudes, and questions about STIs, including attendance at sexual health clinics, previous STI diagnoses, previous STI or HIV tests, STI symptoms, and HPV vaccination. Whenever possible, questions used were consistent between all three Natsal studies, with new or changed questions included after cognitive testing.^17^ However, the wording of the question about clinic attendance was changed in accordance with changes in terminology used in sexual health services. Participants in Natsal-1 and Natsal-2 were asked “Have you ever attended a sexually transmitted disease clinic or special (VD) clinic?” Participants in Natsal-3 were asked “Have you ever attended a sexual health clinic (GUM clinic)?”. Full details of the methods have been described elsewhere.^18^, ^19^, ^20^ An anonymised dataset will be deposited with the UK Data Archive and the complete questionnaire and technical report will be available on the Natsal website on the day of publication.

After the interview, we invited a sample of participants aged 16–44 years (all participants aged 16–17 years; all those aged 18–24 years who reported at least one sexual partner over the lifetime; a random subsample of 85% of 25–44-year-olds who reported at least one sexual partner over the lifetime; and any remaining men aged 25–44 years who reported having sex with another man in the past 5 years) to provide a urine sample for STI testing. We used this strategy to maximise information from groups in whom morbidity and interventions are concentrated, with considerations of sample-size calculations and appropriate use of resources. Urine was collected with the FirstBurst device, which collects the first 4–5 mL of voided urine, thus yielding a higher load of *C trachomatis* than the regular urine cup,^21^ which, on the basis of development work for Natsal-3, might also increase detection of HPV DNA and HIV antibody.^19^ Samples were posted to Public Health England for testing. All participants were given information about where to obtain free diagnostic STI and HIV testing and sexual health advice.

In view of the low population prevalence of some STIs, our predefined testing strategy aimed to reduce the likelihood of false positives. Detection of *C trachomatis* and *N gonorrhoeae* was done with the Aptima Combo 2 assay (Hologic Gen-Probe) as an initial screen, and we confirmed all positive and equivocal results with the Aptima monospecific assays for detection of *C trachomatis* or *N gonorrhoeae*. We used an in-house Luminex-based genotyping assay to detect HPV types.^22^ We defined high-risk types as 16, 18, 31, 33, 35, 39, 45, 51, 52, 56, 58, 59, and 68.^23^ We identified HIV infection with a modified IgG antibody-capture particle-adherence test (GACPAT)^24^ to detect HIV-1 and HIV-2 antibodies in urine;^25^ we confirmed results with HIV western blot 2.2 (MP Biomedicals, UK).

### Statistical analysis

We did all statistical analyses with Stata (version 12.1), accounting for stratification, clustering, and weighting of the sample. We included an additional weight, derived from a logistic regression model, which corrected for unequal probabilities of urine-sample selection, and differential sample response.^19^ Generally, before weighting, younger individuals; those who had had same-sex relationships; and those who engaged in high-risk behaviours, such as more partners with whom they had unprotected sex, were more likely to provide a urine sample than were other participants. We present prevalence estimates in women and men, by age group, with 95% CIs, in participants who reported at least one sexual partner over the lifetime. We examined the associations between chlamydia and high-risk HPV and demographic and behavioural variables with logistic regression and present crude odds ratios (ORs) and adjusted ORs (AORs). Multivariable analyses adjusted for two demographic variables (age and area-level deprivation [index of multiple deprivation; IMD])^26^ and one behavioural factor (number of sexual partners in the past year; a key factor in STI epidemiology and a useful indicator for sexual health-care providers). We considered IMD to be an important predictor and possible confounder, because services and interventions are often commissioned and provided on an area-level basis. We present uptake of interventions by risk factors or target groups, in the relevant age ranges of participants aged 16–44. We compared these findings, when possible, across the three surveys. We estimated the annual rate of chlamydia diagnosis per 100 000 population (an indicator in the Public Health Outcomes Framework for England)^27^ from self-reported chlamydia diagnoses in the past year in all participants aged 16–24 years living in England. We report coverage of HPV vaccination in women who reported any sexual experience and were eligible for the HPV catch-up immunisation programme (ie, were born between Sept 1, 1990, and Aug 31, 1995). We obtained ethics approval from Oxfordshire Research Ethics Committee A (reference 09/H0604/27). Participants gave written informed consent to anonymised testing, without the return of results, the ethical rationale for which has been previously described.^28^ Details about the preparation, testing, and quality assurance of urine samples have been published elsewhere.^18^, ^19^

### Role of funding source

The sponsors of the study had no role in study design, data collection, data analysis, data interpretation, or writing of the report. The corresponding author had full access to all the data in the study and had final responsibility for the decision to submit for publication.

## Results

Of 9902 participants aged 16–44 years, 8947 (90%) reported at least one sexual partner over the lifetime. Of these individuals, 8047 (90%) were invited to provide a urine sample of whom 4828 (60%) agreed. We excluded 278 samples (on the basis of insufficient samples, mislabelling, or unrecorded consent), leaving 4550 (94%) participants with STI test results (2665 women and 1885 men).

98 participants (62 women and 36 men) tested positive for chlamydia. Table 1 shows the weighted age-specific prevalences in women and men. For women, the highest prevalence was in those aged 18–19 years (table 1). By contrast, for men, the highest prevalence was in those aged 20–24 years (table 1). We detected no positive chlamydia tests in men aged 16–17 years and few positives in those aged 18–19 years (table 1). Individuals living in the most deprived regions were more likely to test positive for chlamydia (table 2). Prevalence of chlamydia increased with increasing numbers of partners in the past year (table 2). However, an estimated 60·4% (95% CI 45·5–73·7%) of prevalent chlamydia in women, and 43·3% (25·9–62·5%) in men, was in those with only one partner in the past year. Reporting of two or more partners without use of a condom in the past year was more strongly associated with chlamydia than were partner numbers alone (table 2).

**Table 1:** Prevalence of *Chlamydia trachomatis*, human papillomavirus, and *Neisseria gonorrhoeae* in urine in participants aged 16–44 years, by age group and sex

		**Age 16–17 years**	**Age 18–19 years**	**Age 20–24 years**	**Age 25–34 years**	**Age 35–44 years**	**All ages**
**Women**							
*Chlamydia trachomatis*		2·3% (0·9–5·8)	4·7% (2·5–8·6)	2·7% (1·7–4·2)	1·5% (0·9–2·5)	0·3% (0·1–1·3)	1·5% (1·1–2·0)
Human papillomavirus*							
	High–risk types	16·3% (11·1–23·4)	29·6% (23·5–36·5)	26·6% (22·8–30·8)	15·6% (13·4–18·2)	9·3% (7·1–12·2)	15·9% (14·4–17·5)
	Types 16 or 18	1·2% (0·3–4·6)	6·3% (4·0–9·9)	6·9% (5·1–9·4)	4·7% (3·4–6·3)	2·6% (1·5–4·5)	4·2% (3·4–5·2)
*Neisseria gonorrhoeae*		0·0%	0·0%	0·2% (0·1–0·7)	0·0%	0·0%	<0·1 (0·0–0·1)
**Men**							
*Chlamydia trachomatis*		0·0%	0·5% (0·1–2·2)	3·4% (2·2–5·2)	1·0% (0·5–1·9)	0·3% (0·0–2·1)	1·1% (0·7–1·6)
Human papillomavirus*							
	High-risk types	4·5% (1·9–10·3)	4·0% (2·0–7·9)	8·6% (6·4–11·7)	9·2% (6·6–12·6)	8·8% (5·8–13·3)	8·4% (6·8–10·4)
	Types 16 or 18	0·6% (0·1–4·0)	0·9% (0·2–3·5)	2·7% (1·6–4·7)	2·6% (1·7–4·1)	2·2% (1·1–4·5)	2·3% (1·7–3·2)
*Neisseria gonorrhoeae*		0·0%	0·0%	0·1% (0·0–0·6)	0·0%	0·0%	<0·1 (0·0–0·1)
**Denominator**†**(unweighted, weighted)**							
Women‡		171, 84	224, 130	597, 383	1146, 809	527, 878	2665, 2284
Men‡		150, 91	193, 143	497, 391	693, 807	352, 835	1885, 2266

Data are % (95% CI), unless otherwise indicated.

* Detection rates of human papillomavirus in urine are lower in men than women.

† Denominators shown are for *Chlamydia trachomatis*; denominators for other infections vary slightly.

‡ Participants who reported at least one partner, with urine test results.

**Table 2:** Risk factors for *Chlamydia trachomatis* in urine in participants aged 16–44 years, by sex

		**Women**				**Men**				**Denominator**†**unweighted, weighted**	
		% (95% CI)	OR	AOR*	95% CI	% (95% CI)	OR	AOR*	95% CI	Women	Men
All ages		1·5% (1·1–2·0)	..	..	..	1·1% (0·7–1·6)	..	..	..	2665, 2284	1885, 2266
Age (years)		..	p=0·0026	p=0·0135	..	..	p=0·0002	p=0·0007	..		
	16–19	3·8% (2·2–6·3)	1·00	1·00	..	0·3% (0·1–1·3)	1·00	1·00	..	395, 214	343, 234
	20–24	2·7% (1·7–4·2)	0·71	0·75	0·36–1·57	3·4% (2·2–5·2)	10·55	11·11	2·42–50·93	597, 383	497, 391
	25–34	1·5% (0·9–2·5)	0·40	0·45	0·21–0·97	1·0% (0·5–1·9)	2·97	3·39	0·70–16·40	1146, 809	693, 807
	35–44	0·3% (0·1–1·3)	0·07	0·09	0·02–0·49	0·3% (0·0–2·1)	0·91	1·35	0·10–18·61	527, 878	352, 835
IMD (quintiles)‡		..	p=0·0070	p=0·0078	..	..	p=0·0014	p=0·0028	..		
	1–2 (least deprived)	0·6% (0·2–1·2)	1·00	1·00	..	0·5% (0·2–1·2)	1·00	1·00	..	906, 806	679, 838
	3	1·4% (0·7–2·8)	2·51	2·65	0·92–7·63	0·4% (0·1–1·1)	0·74	0·73	0·18–3·00	521, 458	369, 466
	4–5 (most deprived)	2·2% (1·5–3·2)	4·07	4·01	1·67–9·63	1·9% (1·2–2·9)	3·69	3·42	1·28–9·16	1238, 1021	837, 962
Number of sexual partners in the past year§		..	p=0·0006	P=0·1284	..	..	p<0·0001	p=0·0013	..		
	0–1	1·1% (0·7–1·6)	1·00	1·00	..	0·6% (0·4–1·1)	1·00	1·00	..	2001, 1883	1274, 1731
	2	3·2% (1·7–5·7)	3·03	1·97	0·90–4·31	0·7% (0·2–2·3)	1·09	0·78	0·19–3·15	290, 188	244, 223
	≥3	3·7% (2·1–6·3)	3·53	1·95	0·91–4·20	4·0% (2·2–6·9)	6·42	4·86	1·84–12·80	354, 197	358, 300
Number of sexual partners without a condom in the past year§¶		..	p<0·0001	p=0·0101‖	..	..	p<0·0001	p<0·0001‖	..		
	0‖	1·1% (0·5–2·2)	1·00	1·00	..	0·2% (0·0–0·7)	1·00	1·00	..	469, 428	425, 494
	1	1·1% (0·7–1·7)	1·04	1·10	0·46–2·63	0·7% (0·4–1·2)	4·30	5·96	1·30–27·36	1806, 1629	1111, 1486
	≥2	5·3% (3·3–8·3)	5·10	3·09	1·20–7·96	4·0% (2·4–6·6)	25·34	22·37	5·04–99·24	355, 199	322, 264
Age at first sex (years)**		..	p=0·0717	p=0·8631	..	..	p=0·0997	p=0·7618			
	≥16	1·3% (0·9–1·8)	1·00	1·00	..	0·9% (0·5–1·5)	1·00	1·00	..	1733, 1652	1160, 1520
	<16	2·1% (1·4–3·1)	1·66	1·05	0·62–1·77	1·6% (1·0–2·5)	1·89	1·14	0·50–2·60	901, 608	692, 709
Ever had same-sex experience?††		..	p=0·2412	p=0·1251	..	..	p=0·1272	p=0·0705	..		
	No	1·5% (1·1–2·1)	1·00	1·00	..	1·1% (0·8–1·6)	1·00	1·00	..	2366, 2088	1748, 2144
	Yes	0·8% (0·3–2·3)	0·51	0·40	0·12–1·29	0·2% (0·0–1·7)	0·21	0·15	0·02–1·17	299, 197	137, 121

OR=odds ratio. AOR=adjusted odds ratio. IMD=Index of Multiple Deprivation.

* Adjusted for age, IMD quintiles, and number of sexual partners in the past year.

† Participants who reported at least one partner, with a urine test result.

‡ A multidimensional measure of area (neighbourhood)-level deprivation based on the participant's postcode. We adjusted IMD scores for England, Scotland, and Wales before they were combined and assigned to quintiles, with use of a method by Payne and Abel.^26^

§ Includes both opposite-sex and same-sex partners.

¶ Number of partners without a condom in the past year is adjusted for age and IMD only, because of colinearity with number of sexual partners in the past year.

‖ Includes individuals with no partners in the past year.

** Age at first heterosexual intercourse or first same-sex experience involving genital contact.

†† Same-sex experience involving genital contact.

Prevalence of chlamydia in participants aged 16–24 years—ie, the age group targeted by the NCSP—was 3·1% (2·2–4·3%) in women and 2·3% (1·5–3·4%) in men. Women aged 16–24 years were more likely to have reported a chlamydia test in the past year than men (adjusted OR 2·55, 95% CI 2·13–3·07; table 3). The proportions of women and men tested in England were significantly higher than those tested in Scotland or Wales (table 3). Proportions of individuals testing did not differ by area-level deprivation (table 3). Participants with higher numbers of partners in the past year were more likely to report testing than were those with low partner numbers, as were those with a new partner in the past year (data not shown). However, of individuals aged 16–44 years who had detectable chlamydia in their urine most had not had a chlamydia test (66·4%, 95% CI 55·1%–76·1%), or attended a sexual health clinic (78·7%, 68·3%–86·4%) in the past year.

**Table 3:** Uptake of sexual health interventions and services, by sex

		**Women**				**Men**				**Denominator (unweighted, weighted)**	
		% (95% CI)	Crude OR	AOR*	95% CI	% (95% CI)	Crude OR	AOR*	95% CI	Women	Men
**Reported testing for *Chlamydia trachomatis* in the past year in people aged 16–24 years**†											
All aged 16–24 years		54·2% (51·4–56·9)	..	..	..	34·6% (31·8–37·4)	..	..	..	1736, 966	1375, 1003
By age group (years)		..	p=0·1637	p=0·7670	..	..	p=0·0019	p=0·0023	..		
	16–19	56·6% (52·5–60·6)	1·00	1·00	..	40·4% (35·8–45·1)	1·00	1·00	..	672, 343	582, 374
	20–24	52·8% (49·3–56·3)	0·86	0·97	0·77–1·21	31·1% (27·8–34·7)	0·67	0·67	0·52–0·87	1064, 623	793, 629
By IMD quintile‡		..	p=0·9938	p=0·9492	..	..	p=0·8863	p=0·8303	..		
	1–2 (least deprived)	54·2% (49·7–58·7)	1·00	1·00	..	34·5% (30·1–39·2)	1·00	1·00	..	595, 338	509, 369
	3	54·4% (48·0–60·7)	1·01	1·05	0·76–1·44	33·3% (27·4–39·9)	0·95	0·90	0·62–1·29	324, 189	263, 183
	4–5 (most deprived)	54·0% (49·9–58·1)	0·99	1·00	0·78–1·28	35·2% (31·2–39·4)	1·03	0·98	0·74–1·30	817, 439	603, 450
By number of sexual partners in the past year§		..	p<0·0001	p<0·0001	..	..	p<0·0001	p<0·0001	..		
	0–1¶	46·6% (43·3–49·9)	1·00	1·00	..	26·0% (22·7–29·6)	1·00	1·00	..	1096, 624	768, 568
	2	65·2% (59·0–70·9)	2·15	2·13	1·58–2·86	40·3% (33·2–47·7)	1·92	1·88	1·32–2·67	275, 143	251, 185
	≥3	71·8% (65·4–77·4)	2·91	2·88	2·07–4·02	50·8% (44·8–56·8)	2·93	2·87	2·11–3·91	345, 188	340, 237
By country		..	p<0·0001	p<0·0001	..	..	p=0·0001	p<0·0001	..		
	England	57·1% (54·1–60·1)	1·00	1·00	..	37·3% (34·3–40·3)	1·00	1·00	..	1452, 823	1181, 859
	Scotland	32·4% (24·2–41·8)	0·36	0·34	0·23–0·51	22·2% (13·6–34·1)	0·48	0·44	0·24–0·82	178, 91	111, 89
	Wales	45·6% (36·2–55·3)	0·63	0·60	0·38–0·96	12·8% (6·7–23·0)	0·25	0·21	0·10–0·44	106, 52	83, 55
**Reported completion of a three-dose course of HPV vaccination in participants eligible for the catch–up programme**‖											
All eligible		61·5% (58·2–64·7)	..	..	..	..	..	..	..	1050, 562	..
By IMD quintile‡		..	p<0·0001	p=0·0001	..	..	..	..	..	..	..
	1–2 (least deprived)	69·9% (64·6–74·7)	1·00	1·00	..	..	..	..	..	393, 210	..
	3	63·3% (55·4–70·6)	0·74	0·71	0·47–1·07	..	..	..	..	209, 116	..
	4–5 (most deprived)	53·1% (48·1–58·0)	0·49	0·50	0·36–0·68	..	..	..	..	448, 236	..
By number of sexual partners over the lifetime§		..	p<0·0001	p=0·0090	..	..	..	..	..	..	
	0	75·5% (68·3–81·5)	1·00	1·00	..	..	..	..	..	205, 109	..
	1	65·6% (57·5–72·8)	0·62	0·69	0·42–1·15	..	..	..	..	203, 113	..
	2	62·5% (53·3–70·8)	0·54	0·63	0·37–1·08	..	..	..	..	147, 77	..
	≥3	53·8% (48·9–58·7)	0·38	0·47	0·30–0·74	..	..	..	..	488, 260	..
Reported testing for HIV in the past 5 years**											
All aged 16–44 years		27·6% (26·1–29·1)	..	..	..	16·9% (15·5–18·5)	..	..	..	4967, 3458	3429, 3502
By age group (years)		..	p<0·0001	p<0·0001	..		p<0·0001	p<0·0001	..		
	16–24	29·3% (26·9–31·8)	1·00	1·00	..	14·0% (12·0–16·2)	1·00	1·00		1636, 911	1315, 958
	25–34	36·1% (33·8–38·5)	1·36	1·87	1·59–2·21	24·3% (21·7–27·1)	1·98	2·85	2·24–3·62	2236, 1235	1372, 1229
	35–44	18·4% (16·0–21·0)	0·54	0·88	0·71–1·09	12·2% (9·9–14·9)	0·86	1·69	1·22–2·34	1095, 1312	742, 1315
By IMD quintile‡		..	p=0·0096	p=0·0096	..	..	p=0·3794	p=0·6897	..		
	1–2 (least deprived)	24·7% (22·4–27·0)	1·00	1·00	..	15·9% (13·6–18·6)	1·00	1·00		1708, 1257	1241, 1299
	3	26·8% (23·7–30·2)	1·12	1·14	0·93–1·40	16·1% (13·4–19·2)	1·01	1·04	0·78–1·40	957, 683	656, 681
	4–5 (most deprived)	30·4% (28·2–32·6)	1·33	1·29	1·10–1·52	18·1% (15·9–20·6)	1·17	1·12	0·87–1·44	2302, 1518	1532, 1523
By number of sexual partners in the past 5 years§		..	p<0·0001	p<0·0001	..	..	p<0·0001	p<0·0001	..		
	0–1	21·8% (20·0–23·7)	1·00	1·00	..	9·1% (7·5–11·0)	1·00	1·00	..	2493, 1981	1372, 1713
	2–4	30·3% (27·7–33·0)	1·56	1·47	1·24–1·75	17·0% (14·4–19·8)	2·03	2·31	1·72–3·10	1520, 926	1055, 960
	5–9	44·8% (40·1–49·6)	2·91	2·67	2·12–3·37	26·4% (22·2–31·0)	3·57	4·27	3·06–5·94	589, 343	538, 442
	≥10	49·2% (42·6–55·8)	3·48	3·11	2·30–4·20	41·6% (36·4–47·0)	7·09	8·49	6·12–11·76	313, 167	435, 358
Among target groups††											
People who attended a GUM or sexual health clinic in the past 5 years		58·3% (55·3–61·4)	..	..	..	50·8% (46·4–55·1)	..	..	..	1270, 737	811, 676
	Those diagnosed with an STI in the past 5 years‡‡	68·3% (62·4–73·6)	..	..	..	54·7% (47·1–62·1)	..	..	..	376, 210	243, 214
	Women who had attended antenatal services in the past 5 years	47·9% (44·7–51·0)	..	..	..	..	..	..	..	1326, 872	..
	Women who had had an abortion in the past 5 years§§	48·8% (42·5–55·2)	..	..	..	..	..	..	..	310, 164	..
	Men who had had sex with a man in the past 5 years	..	..	..	..	51·6% (41·1–61·9)	..	..	..	..	134, 112
	People of black African ethnic origin	46·1% (35·6–57·0)	..	..	..	43·9% (30·3–58·6)	..	..	..	96, 75	71, 82
Reported attendance at sexual health clinics in the past 5 years**											
All aged 16–44 years		21·4% (20·1–22·7)	..	..	..	19·6% (18·2–21·2)	..	..	..	5234, 3651	3546, 3624
By age group (years)		..	p<0·0001	p<0·0001	..		p<0·0001	p<0·0001	..		
	16–24	43·8% (41·1–46·5)	1·00	1·00	..	31·4% (28·6–34·4)	1·00	1·00	..	1701, 947	1353, 987
	25–34	21·3% (19·4–23·4)	0·35	0·55	0·46–0·66	23·6% (21·3–26·1)	0·67	0·97	0·79–1·22	2365, 1306	1418, 1269
	35–44	6·3% (5·0–7·8)	0·09	0·19	0·14–0·25	7·5% (5·8–9·7)	0·18	0·37	0·26–0·51	1168, 1398	775, 1368
By IMD quintile‡			p<0·0001	p=0·0007	..		p=0·0184	p=0·1682	..		
	1–2 (least deprived)	18·3% (16·5–20·3)	1·00	1·00	..	17·0% (14·8–19·5)	1·00	1·00	..	1791, 1327	1279, 1338
	3	19·9% (17·3–22·9)	1·12	1·10	0·86–1·40	20·2% (17·1–23·7)	1·22	1·25	0·94–1·66	1022, 726	682, 709
	4–5 (most deprived)	24·6% (22·7–26·6)	1·45	1·41	1·18–1·70	21·6% (19·5–23·9)	1·36	1·22	0·97–1·54	2421, 1598	1585, 1576
By number of sexual partners in the past 5 years§		..	p<0·0001	p<0·0001	..		p<0·0001	p<0·0001	..		
	0–1	7·7% (6·7–8·9)	1·00	1·00	..	5·8% (4·8–7·1)	1·00	1·00	..	2644, 2113	1439, 1795
	2–4	30·5% (28·0–33·2)	5·24	3·77	3·09–4·59	24·3% (21·5–27·4)	5·20	4·12	3·11–5·44	1625, 992	1093, 1001
	5–9	55·1% (50·4–59·7)	14·66	8·88	6·88–11·46	34·9% (30·3–39·9)	8·69	6·37	4·64–8·74	621, 359	554, 453
	≥10	65·6% (59·2–71·4)	22·73	12·11	8·65–16·96	55·9% (50·6–61·1)	20·54	15·36	11·15–21·16	320, 171	441, 359
In people diagnosed with an STI in the past 5 years‡‡		83·7% (79·1–87·4)	..	..	..	85·4% (79·4–89·8)	..	..	..	322, 220	213, 215
In men who had had sex with a man in the past 5 years		..	..	..	..	45·0% (35·0–55·5)	..	..	..	..	136, 115

OR=odds ratio. AOR=adjusted odds ratio. IMD=Index of Multiple Deprivation. GUM=genitourinary medicine.

* ORs adjusted for age, adjusted IMD, and number of sexual partners in the past 1 or 5 years.

† Denominator is individuals aged 16–24 years who reported at least one partner over the lifetime.

‡ A multidimensional measure of area (neighbourhood)-level deprivation based on the participant's postcode. We adjusted IMD scores for England, Scotland, and Wales before they were combined and assigned to quintiles, with use of a method by Payne and Abel.^26^

§ Includes both opposite-sex and same-sex partners.

¶ Testing was lower in individuals with no partners than in those who had had one partner in the past year: 21·5% (95% CI 11·2–37·4) *vs* 47·9% (44·4–51·3) for women and 5·7% (2·3 −13·7) *vs* 28·0% (24·4 −31·9) for men. Because the unweighted denominators for individuals with no partners were small (48 women and 73 men), we combined the 0 and 1 categories.

‖ Denominator is women eligible for human papillomavirus vaccination catch-up programme—ie, those born between Sept 1, 1990, and Aug 31, 1995 who reported some sexual experience.

** Denominator is participants aged 16–44 years who reported at least one partner over the lifetime.

†† Target groups identified in UK National Guidelines for HIV Testing 2008.^16^

‡‡ Diagnosed with chlamydia, gonorrhoea, herpes, genital warts, trichomonas, non-specific or non-gonococcal urethritis, or syphilis.

§§ Abortion in the past 5 years was used as a proxy for attending a clinic for termination of pregnancy.

Of participants aged 16–24 years who reported at least one partner over the lifetime, 3·0% (2·2–4·0) of women and 2·0% (1·3–3·0) of men had been diagnosed with chlamydia in the past year. We estimated an annual rate of chlamydia diagnosis of 2016 (95% CI 1545–2627) per 100 000 population aged 16–24 years in England. When we compared Natsal-2 and Natsal-3, we noted large increases in the proportion of participants aged 16–24 years who reported being diagnosed with chlamydia in the past 5 years: from 1·5% (1·2–1·8) to 4·1% (3·6–4·7) in women and from 0·8% (0·5–1·1) to 4·0% (3·4–4·8) in men. However, in Natsal-2 compared with Natsal-3, prevalence of chlamydia in urine in young people aged 18–24 years was broadly similar in women (3·1% [1·8–5·2] *vs* 3·2% [2·2–4·6], and men (2·9% [1·3–6·3] *vs* 2·6% [1·7–4·0]).

High-risk HPV was detected in the urine of 527 (15·9%) women and 164 (8·4%) men (table 1). The weighted age-specific prevalence differed in women and men: from age 20 onwards, prevalence reduced with age in women, but remained stable in men (table 1). The prevalence of HPV types 16 and 18 was roughly a quarter of that of all high-risk HPV (table 1). Increasing numbers of partners without condom use in the past year were associated with high-risk HPV in both women and men (table 4). Prevalence of high-risk HPV in participants aged 18–44 was similar in Natsal-3 and Natsal-2: 15·9% (95% CI 14·3–17·6) *vs* 16·0% (14·2–18·0) in women and 8·6% (6·9–10·7) *vs* 9·9% (8·2–11·9) in men.

**Table 4:** Risk factors for high-risk human papillomavirus in urine among in participants aged 16–44 years, by sex

		**Women**				**Men**				**Denominator**†**unweighted, weighted**	
		% (95% CI)	OR	AOR*	95% CI	% (95% CI)	OR	AOR*	95% CI	Women	Men
All ages		15·9% (14·4–17·5)	..	..	..	8·4% (6·8–10·4)	..	..	..	2569, 2189	1799, 2165
Age (years)		..	p<0·0001	p<0·0001	..	..	p=0·0705	p=0·0404	..	..	..
	16–19	24·4% (20·0–29·3)	1·00	1·00	..	4·2% (2·5–7·1)	1·00	1·00	..	377, 203	326, 222
	20–24	26·6% (22·8–30·8)	1·13	1·38	0·97–1·97	8·6% (6·4–11·7)	2·16	2·20	1·15–4·17	580, 370	475, 371
	25–34	15·6% (13·4–18·2)	0·58	0·83	0·59–1·18	9·2% (6·6–12·6)	2·30	2·44	1·27–4·70	1108, 779	661, 766
	35–44	9·3% (7·1–12·2)	0·32	0·53	0·34–0·80	8·8% (5·8–13·3)	2·21	2·46	1·19–5·10	504, 837	337, 805
IMD (quintiles)‡		..	p=0·0238	p=0·0508	..	..	p=0·5621	p=0·5781	..	..	..
	1–2 (least deprived)	13·5% (11·2–16·1)	1·00	1·00	..	7·6% (5·5–10·5)	1·00	1·00	..	873, 778	653, 808
	3	15·0% (11·8–18·7)	1·13	1·16	0·81–1·64	7·6% (4·2–13·3)	0·99	0·99	0·48–2·04	502, 439	356, 452
	4–5 (most deprived)	18·3% (15·9–20·9)	1·43	1·40	1·07–1·84	9·6% (7·0–13·0)	1·28	1·28	0·78–2·10	1194, 973	790, 904
Number of sexual partners in the past year§		..	p<0·0001	p<0·0001	..	..	p=0·3639	p=0·2841	..	..	..
	0–1	12·1% (10·6–13·7)	1·00	1·00	..	8·0% (6·1–10·5)	1·00	1·00	..	1925, 1802	1213, 1651
	2	26·1% (20·3–32·9)	2·57	2·18	1·51–3·14	8·3% (5·2–13·0)	1·05	1·13	0·62–2·07	276, 179	237, 217
	≥3	41·2% (35·1–47·7)	5·11	3·95	2·87–5·45	11·1% (7·6–15·9)	1·44	1·53	0·90–2·58	349, 194	340, 285
Number of sexual partners without a condom in the past year§¶		..	p<0·0001	p<0·0001‖	..	..	p=0·0219	p=0·0141‖	..	..	..
	0‖	11·3% (8·5–14·9)	1·00	1·00	..	5·2% (2·8–9·6)	1·00	1·00	..	451, 408	407, 473
	1	14·0% (12·3–15·8)	1·27	1·32	0·94–1·87	8·8% (6·7–11·4)	1·73	1·65	0·78–3·47	1739, 1564	1060, 1418
	≥2	40·1% (34·0–46·5)	5·23	4·24	2·79–6·44	13·4% (9·3–19·0)	2·80	2·88	1·36–6·11	347, 193	305, 251
Age at first sex (years)**		..	p<0·0001	p=0·1580	..		p=0·2137	p=0·2569	..	..	..
	≥16	14·2% (12·5–16·2)	1·00	1·00	..	7·7% (5·7–10·4)	1·00	1·00	..	1665, 1577	1105, 1451
	<16	20·9% (18·1–24·1)	1·60	1·20	0·93–1·55	10·0% (7·6–13·0)	1·32	1·29	0·83–2·00	874, 587	663, 678
Ever had same-sex experience?††		..	p=0·0309	p=0·6037	..	..	p=0·3272	p=0·1578	..	..	..
	No	15·4% (13·8–17·1)	1·00	1·00	..	8·6% (6·9–10·7)	1·00	1·00	..	2277, 1998	1672, 2052
	Yes	21·2% (16·2–27·3)	1·48	1·11	0·74–1·66	6·0% (2·9–11·8)	0·67	0·55	0·24–1·26	292, 192	127, 113

OR=odds ratio. AOR=adjusted odds ratio. IMD=Index of Multiple Deprivation.

* Adjusted for age, IMD quintiles, and number of sexual partners in the past year.

† Participants who reported at least one partner, with a urine test result.

‡ A multidimensional measure of area (neighbourhood)-level deprivation based on the participant's postcode· We adjusted IMD scores for England, Scotland, and Wales before they were combined and assigned to quintiles, with use of a method by Payne and Abel.^26^

§ Includes both opposite-sex and same-sex partners.

¶ Number of partners without a condom in the past year is adjusted for age and IMD only, because of colinearity with number of sexual partners in the past year.

‖ Includes individuals with no partners in the past year.

** Age at first heterosexual intercourse or first same-sex experience involving genital contact.

†† Same-sex experience involving genital contact.

Of women eligible for the HPV catch-up immunisation programme, 61·5% reported completing the three-dose vaccination course (table 3). Vaccination coverage was lowest in individuals from the most deprived areas and in those with more partners (table 3). Prevalence of HPV types 16 and 18 in women aged 18–20 years was 5·8% (95% CI 3·9–8·6), which was lower than that noted before introduction of the immunisation programme in Natsal-2 (11·3% [6·8–18·2]; age-adjusted OR 0·44 [0·21–0·94]). Prevalence of HPV types 16 or 18 was likewise reduced in men in Natsal-3 (1·1% [0·4–3·0]) compared with those in Natsal-2 (5·0% [1·7–13·6]; age-adjusted OR 0·20 [0·04–0·93]).

Three women and one man had urine tests that were positive for gonorrhoea, giving a weighted prevalence of less than 0·1% in both women and men aged 16–44 years (table 1). These participants were all aged 20–24 years and reported sex with at least two partners in the past year without use of condoms. All four had chlamydia co-infection.

Three women and six men tested positive for HIV antibody, giving estimated prevalences of 0·1% (95% CI 0·0–0·4) in women and 0·2% (0·1–0·6) in men. Of these participants, five were white British men who reported having had sex with a man over the lifetime, giving an estimated weighted prevalence of 2·8% (1·1–6·9) in this group. The three women and one man who did not report any same-sex experiences and tested HIV positive were all of black ethnic origin; the prevalence estimate in this group was 2·8% (1·0–7·7). All eight participants who answered the question reported having had an HIV test in the past 5 years and receiving the result. None of the HIV-positive participants reported a history of injecting drug use.

**Figure 1:**
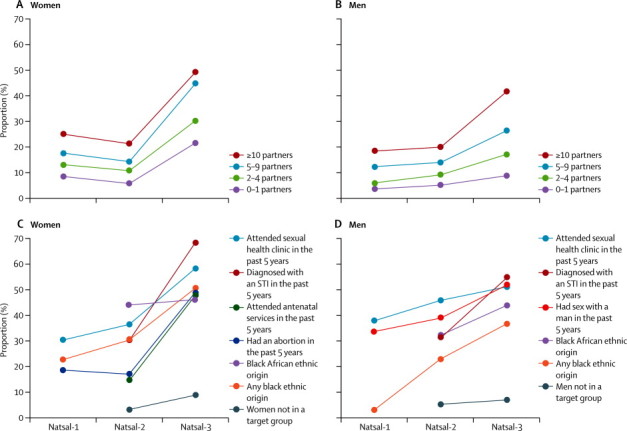
Change over time in reported HIV testing in the past 5 years, by number of sexual partners (A, B) and target group (C, D) Denominator is individuals aged 16–44 years who reported at least one sexual partner over the lifetime. The appendix shows the proportions, 95% CIs, and p values for the difference between Natsal-3 and Natsal-2.

More women than men reported HIV testing in the past 5 years (table 3). Testing was highest in individuals aged 25–34 years, in those with more sexual partners, in those who had attended a sexual health clinic, in those who had been diagnosed with an STI in the past 5 years, in people of black African ethnic origin, and in men who had sex with men in the past 5 years. The proportion of men who had sex with men who had HIV tests increased with increasing partner numbers (data not shown), reaching 84·5% (95% CI 69·4–92·9) in men reporting five or more male partners in the past 5 years. The proportion of participants reporting having had an HIV test in the past 5 years was higher in Natsal-3 (27·6% in women and 16·9% in men; table 3) than in Natsal-2 (8·7% *vs* 9·2%) and Natsal-1 (10·5% *vs* 6·6%; appendix). HIV testing was generally higher with increasing numbers of sexual partners and in groups specifically targeted for testing (figure 1), with increases in testing mostly occurring in the past 10 years.

**Figure 2:**
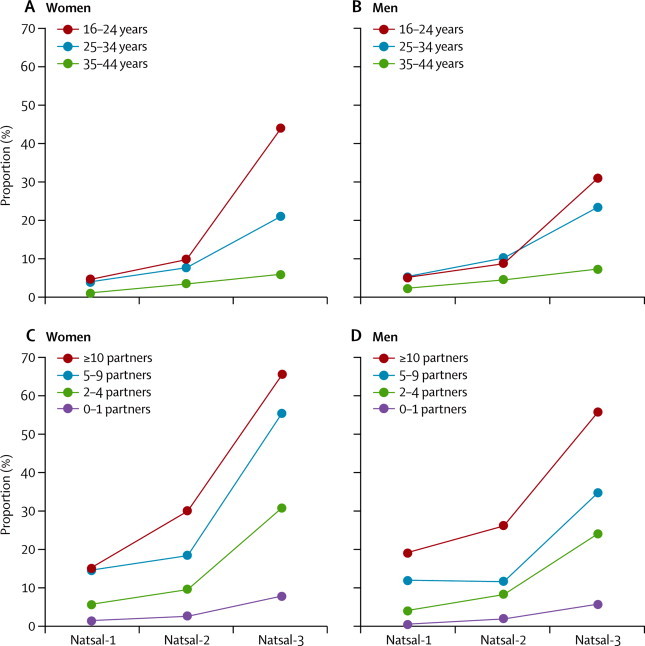
Change over time in reported attendance at sexual health clinics in the past 5 years, by age group (A, B) and number of partners (C, D) Denominator is individuals aged 16–44 years who reported at least one sexual partner over the lifetime. The appendix shows the proportions, 95% CIs, and p values for the difference between Natsal-3 and Natsal-2.

In the past 5 years, roughly a fifth of women and men reported attendance at a sexual health clinic, with attendance highest in the youngest age group, in those with higher numbers of partners, and in women from the most deprived areas (table 3). More than 80% of participants reporting an STI diagnosis in the past 5 years had attended a sexual health clinic (table 3). Figure 2 shows attendance at sexual health clinics in the past 5 years in women and men aged 16–44 years across the three Natsal surveys, by number of partners and age group. We noted a significant increase in clinic attendance over the three surveys, with increasing attendance seen in all subgroups (appendix).

## Discussion

Findings from this large population-based survey show that the four STIs are distributed heterogeneously in the British population. High-risk HPV was the most prevalent infection, followed by chlamydia; HIV and gonorrhoea were uncommon. Although STI risk increased with increasing partner numbers, most of the chlamydia and HPV infections were in individuals who did not have many recent partners because most of the population had only had one partner in the past year.^20^ For chlamydia and HPV, broad population-wide interventions are needed. By contrast, we show that gonorrhoea and HIV were restricted to a small proportion of the population who had high risk factors, including other STIs, supporting targeted interventions. STI transmission is a function of individual and partnership risks, as shown by the sex differences in the age distribution of chlamydia, which is related to patterns of sexual mixing, because younger women, on average, have older male partners.^29^

STI-specific interventions that take account of the epidemic phase^30^ are key components of STI prevention strategies. More focused interventions that include outbreak investigation and contact tracing might be more cost-effective than generalised screening for rare infections. The very low population prevalence of gonorrhoea allays concerns about widespread or asymptomatic infection in the community. The prevalence of gonorrhoea was substantially higher in people testing positive for chlamydia than it was in the general population, suggesting that, in addition to outbreak investigation, gonorrhoea testing in people with chlamydia identified through population screening might be an appropriate strategy.

Survey participants and those providing urine might not be fully representative of the general population, despite our best efforts to adjust for known biases. To minimise non-participation bias, we weighted Natsal-3 data to correct for differential selection probabilities and for differential non-response (by comparing with census data).^18^, ^19^, ^20^ Application of the urine selection and non-response weights further reduced participation bias. As discussed elsewhere,^18^, ^20^ to minimise reporting bias, we included an extensive development phase^17^ and used computer-assisted self-interview for sensitive questions.

In this population-based study, we undertook tests on urine rather than genital specimens for practicality and acceptability. We recognise that urine is a suboptimum specimen for the detection of some STIs, particularly HPV in men, and that prevalence might be underestimated, although we are able to assess risk factors. Comparisons of urine prevalence estimates between Natsal-2 and Natsal-3 should be made with caution, in view of differences in methods of sample collection, preparation and storage, chlamydia diagnostic tests, and because the surveys were not powered to detect changes in prevalence. Trends in STI prevalence should be considered in the context of possible changes in sexual behaviour over time. Between Natsal-2 and Natsal-3, modest increases in some risk behaviours have taken place (including increases in partner numbers) in women, but evidence shows some reduction in men, by contrast with the increases in both sexes noted between Natsal-1 and Natsal-2.^20^

Compared with Natsal-2, which tested urine from a sample of participants aged 18–44 years, in Natsal-3 we extended the age range to include 16–17-year-olds. This change improved prevalence estimates in the younger age groups, who have the highest prevalence and greatest risk of sequelae and are therefore the age groups at whom interventions are targeted. People remain sexually active into older age,^20^ including having new partners, which means that STI risk continues. Surveillance data show that the rate of acute STIs diagnosed in sexual health clinics in individuals aged 20–24 years is almost 20 times higher than that in those aged 44–59 years (4278 per 100 000 population *vs* 227 per 100 000 population in 2012).^2^ Although recent reports have shown increases in some diagnosed STIs in older age groups, the absolute rates are very low.^2^ Therefore, even a population-based survey of more than 15 000 participants, as in Natsal-3, would not have sufficient power to estimate STI prevalence in those older than 44 years with useful precision.

Attendance at sexual health clinics and uptake of HIV testing have increased substantially in the past two decades. Furthermore, many sexually active young adults reported having had a chlamydia test in the past year. Although it is reassuring that large and increasing proportions of the population at highest risk are accessing services and testing, many people have still not done so. For example, two-thirds of the participants in whom chlamydia was detected in our study did not report a test in the past year, and more than three-quarters had not attended a sexual health service in the past year. Roughly half of the sexually active men who had sex with men in our study had tested for HIV in the past 5 years and only 27% (95% CI 19–37) in the past year—far less than the recommendation to test all men in this group annually.^16^ Convenience surveys of men who have sex with men have reported that 50–60% of men have been tested in the past year,^31^, ^32^ whereas surveillance data estimate about 10%.^33^ Our results provide a valuable population-based estimate by including men who have sex with men who do not necessarily identify as gay or use sexual health services.

One in five sexually active participants reported attendance at a sexual health clinic in the past 5 years, which is consistent with estimates from clinic surveillance data (overall 19·9% of women and 17·2% of men, with age-specific proportions also similar).^34^ This finding suggests that a minimum amount of misclassification bias took place when individuals answered the revised question in Natsal-3, despite the broad range of settings used for sexual health services in 2010. 80% of people with an STI diagnosis in the past 5 years reported attendance at a sexual health clinic, suggesting that in most cases, treatment for STIs occurs in specialist services. Uptake of antenatal HIV testing, measured from unlinked anonymous surveillance, was 97% in 2011;^33^ however, the reporting of this testing was much lower in Natsal-3 (48%). Although this proportion is significantly higher than the 15% reported in Natsal-2, both figures probably show women's low awareness or poor recall of having been tested, in the context of a large variety of screening tests in an antenatal clinic.

Reducing inequalities is a key principle of the National Strategy for Sexual Health and HIV.^13^ Measurement of inequalities is complex. Different indicators might result in different findings, as shown in analyses of sexual behaviours and attitudes by area-level deprivation, educational attainment, and National Statistics Socio-economic Classification (NS-SEC) in participants aged 16–74 years in Natsal-3.^20^ Similarly, a review examining the association between socioeconomic circumstances and chlamydia prevalence in various countries showed substantial variations, dependent on the measure used.^35^ The IMD at small area level has seven domains (income, employment, health, education, housing and services, crime, and living environment).^26^ We used this index as a multidimensional measure of social deprivation, in recognition that individual-level indicators, such as NS-SEC, can be difficult to define and interpret, especially in young people. We recorded a strong association between area-level deprivation and chlamydia prevalence, but no association with chlamydia testing uptake, suggesting that increases in the levels and intensity of testing in the most deprived areas might be needed.

The rise in chlamydia diagnoses, as shown in surveillance data,^2^ is better explained by increases in ascertainment through increased service use and testing than by increases in sexual risk behaviour.^20^ Although the estimated rate of chlamydia diagnoses is approaching the Public Health Outcomes Framework target of 2300 per 100 000 population in England,^27^ increased chlamydia testing is needed. In view of the levels of chlamydia testing, the increase in diagnoses, and estimates from mathematical models,^36^ we might expect to see reductions in chlamydia prevalence. Comparisons between prevalence estimates from Natsal-2^5^ and Natsal-3 need to consider the caveats mentioned above. Present prevalence might have been higher without improved chlamydia control.

In both Natsal-2^6^ and Natsal-3, the prevalence of high-risk HPV was higher in women than men in all age groups partly due to the lower sensitivity of urine-based testing to identify true genital infection with HPV in men compared with women.^37^ The reported uptake of HPV vaccination in participants eligible for the catch-up programme was more than 60%; however, coverage was lower in groups at higher risk. We were unable to assess uptake of the routine programme (in girls aged 12–13 years), because very few were old enough to be included in Natsal-3 by 2012. However, estimated coverage from programme data has been more than 80%, with no significant variation by area-level deprivation,^38^ although uptake needs ongoing monitoring. Reduction in the prevalence of high-risk HPV types targeted by the vaccine is an early measure of the effect of the HPV immunisation programme. We noted a reduction in the prevalence of HPV types 16 and 18 in young women that was of similar magnitude to that reported in recent surveillance data.^39^ We also recorded a reduction in these HPV types in young men. These findings need to be explored in the context of the changes in sample collection, other HPV types, patterns of sexual mixing, behaviour, and herd immunity. In September, 2012, the UK programme switched from the bivalent to the quadrivalent vaccine, which includes HPV types 6, 11, 16, and 18. Data from Australia and the USA, with use of the quadrivalent vaccine, have shown reductions in all four of these HPV types in women in the target age groups,^40^, ^41^ and, in a further measure of the effect of the quadrivalent vaccine, in the incidence of genital warts.^42^

National guidelines recommend expansion of HIV testing for all individuals admitted to hospital, and in general practice, in regions where diagnosed HIV prevalence is greater than 2 per 1000 population (ie, 0·2%) in 15–59-year-olds.^16^ Although the overall HIV prevalence was less than this threshold, the prevalence in some groups, such as men who have sex with men and those of black ethnic origin, was estimated to be more than ten times higher than the overall population prevalence. Too few people had HIV infection to provide region-specific estimates of prevalence. Although the finding that eight of the nine HIV-positive participants in Natsal-3 reported previous testing is reassuring, we cannot know at what stage in their infection, or whether, they were diagnosed. National surveillance data show that many people in the community have undiagnosed infection, such that many present with late-stage disease.^33^ The HIV-positive participants in Natsal-3 all had clearly identifiable risks, reinforcing the need to raise awareness of HIV and its prevention, particularly in the groups most affected. To detect HIV both within and outside the main risk groups, health professionals should take an appropriate history and increase testing in routine clinical practice.

The greatly increased uptake of sexual health services in the past decade, particularly in people at increased STI risk, is encouraging (panel). Three of the interventions we measured are indicators in the Public Health Outcomes Framework^27^ (chlamydia diagnoses rate, HPV immunisation, and HIV testing [late diagnoses]) and are included in the 2013 Framework for Sexual Health Improvement in England.^43^ This framework, and the Sexual Health and Wellbeing Action Plan for Wales 2010–15,^44^ also emphasise the need for high-quality integrated sexual health services and promotion of prevention strategies. At a time of change in the organisation and structure of service delivery and commissioning, these findings provide empirical evidence to inform future sexual health interventions and services.

Panel **Research in context**

**Systematic review**

While many developed countries have national sexual health services and surveillance,^45^ few population-based surveys (the National Health and Nutrition Examination Survey^46^ and AddHealth ^47^ in the USA and Contexte de la Sexualité en France^48^) have included testing for sexually transmitted infections (STIs). Natsal-2 included biological sampling, and tested for *Chlamydia trachomatis*, showing a clear association with sexual behaviour, particularly multiple sexual partnerships,^5^ and provided baseline estimates of type-specific human papillomavirus (HPV) before the introduction of the HPV vaccination programme in 2008.^6^ Many large-scale population-based public health interventions are not subject to randomised controlled trials and can rely on natural experiments ^49^ or mathematical modelling to assess effect.^36^, ^50^ Comparison within populations and across countries is difficult because variations might exist in underlying STI epidemiology, health services, surveillance systems, and STI control measures. The UK has a network of accessible, confidential sexual health clinics, a National Health Service that is free at the point-of-care, and an STI surveillance system that has existed for almost a century. The combination of repeated cross-sectional national probability sexual health surveys, with detailed behavioural data and STI testing, surveillance, and comprehensive free open-access services, is, to our knowledge, unique.

**Interpretation**

Our study updates prevalence estimates and risk factors for chlamydia and HPV, and, for the first time, reports population prevalences for gonorrhoea and HIV. With use of data from all three National Survey of Sexual Attitudes and Lifestyles (Natsal) studies, we show increases in reported chlamydia diagnoses and HIV testing, and in attendance at sexual health clinics, especially in individuals at highest risk. We also report coverage of HPV catch-up programmes and early findings of a reduction in HPV types 16 and 18 in women in the eligible age group. Although Natsal is not specifically designed or powered to evaluate particular interventions or initiatives, especially in subgroups of the population, the surveys allow us to track progress and measure some outcomes to complement and validate routinely collected data. Furthermore, Natsal provides empirical population-based estimates of prevalence and uptake according to risk, and behavioural data to populate mathematical models.

## Supplementary Material

Supplementary appendix

Supplementary video Results of over 45 000 respondents, recording the dramatic changes in our sexual lifestyles over the past 60 years.
