# Supplementary material for: Prevalence, risk factors, and uptake of interventions for sexually transmitted infections in Britain: findings from the National Surveys of Sexual Attitudes and Lifestyles (Natsal)
Source: Lancet. 2013 Nov 30;382(9907):1795–806. doi: 10.1016/S0140-6736(13)61947-9 (PMC3899025; doi:10.1016/S0140-6736(13)61947-9)
Supplement: Supplementary appendix [file mmc1.pdf]

THE LANCET

Supplementary appendix

This appendix formed part of the original submission and has been peer reviewed. We post it as supplied by the authors.

Supplement to: Sonnenberg P, Clifton S, Beddows S, et al. Prevalence, risk factors, and uptake of interventions for sexually transmitted infections in Britain: findings from the National Surveys of Sexual Attitudes and Lifestyles (Natsal). *Lancet* 2013; published online Nov 26. http://dx.doi.org/10.1016/S0140-6736(13)61947-9.

Web appendix: Sexual Health Clinic attendance and HIV testing in participants aged 16-44: by sex and survey year

	WOMEN							MEN						
	Natsal-1		Natsal-2		Natsal-3			Natsal-1		Natsal-2		Natsal-3		
	%	95% C.I.	%	95% C.I.	%	95% C.I.	p-value[10]	%	95% C.I.	%	95% C.I.	%	95% C.I.	p-value[10]
**Reported attendance at sexual Health clinic[1] in the past 5 years**	3·4%	(2·9-4·0)	6·7%	(6·0-7·5)	21·4%	(20·1-22·7)	<0·0001	4·4%	(3·8-5·0)	7·7%	(6·8-8·6)	19·6%	(18·2-21·2)	<0·0001
**By age group (years)**														
16-24	5·0%	(3·8-6·5)	10·1%	(8·4-12·1)	43·8%	(41·1-46·5)	<0·0001	5·2%	(4·0-6·8)	8·9%	(7·1-11·2)	31·4%	(28·6-34·4)	<0·0001
25-34	4·2%	(3·5-5·2)	7·8%	(6·7-9·1)	21·3%	(19·4-23·4)	<0·0001	5·4%	(4·5-6·6)	10·2%	(8·7-11·9)	23·6%	(21·3-26·1)	<0·0001
35-44	1·3%	(0·9-1·8)	3·6%	(2·9-4·5)	6·3%	(5·0-7·8)	0·0005	2·5%	(1·9-3·4)	4·6%	(3·6-5·7)	7·5%	(5·8-9·7)	0·0048
**By number of sexual partners[2] in the past 5 years**														
0-1	1·4%	(1·1-1·9)	2·4%	(1·9-3·0)	7·7%	(6·7-8·9)	<0·0001	0·8%	(0·6-1·2)	2·1%	(1·6-2·9)	5·8%	(4·8-7·1)	<0·0001
2-4	5·7%	(4·5-7·1)	9·3%	(7·9-11·0)	30·5%	(28·0-33·2)	<0·0001	4·2%	(3·2-5·4)	8·2%	(6·6-10·0)	24·3%	(21·5-27·4)	<0·0001
5-9	14·9%	(11·2-19·6)	17·9%	(14·2-22·4)	55·1%	(50·4-59·7)	<0·0001	12·1%	(9·3-15·7)	11·6%	(9·2-14·5)	34·9%	(30·3-39·9)	<0·0001
10+	15·2%	(8·1-26·8)	29·6%	(23·4-36·5)	65·6%	(59·2-71·4)	<0·0001	19·2%	(15·1-24·0)	26·2%	(22·2-30·7)	55·9%	(50·6-61·1)	<0·0001
**Denominators[3,4,5]**	7090, 6117		6005, 5277		5234, 3651			5402, 6184		4384, 5101		3546, 3624		
**Tested for HIV, past 5 years**	10·5%	(9·7-11·4)	8·7%	(7·9-9·6)	27·6%	(26·1-29·1)	<0·0001	6·6%	(5·9-7·4)	9·2%	(8·3-10·2)	16·9%	(15·5-18·5)	<0·0001
**By age group (years)**														
16-24	11·6%	(9·9-13·6)	9·4%	(7·6-11·6)	29·3%	(26·9-31·8)	<0·0001	6·3%	(4·9-8·2)	7·2%	(5·7-9·0)	14·0%	(12·0-16·2)	<0·0001
25-34	13·4%	(12·1-15·0)	11·3%	(9·9-12·9)	36·1%	(33·8-38·5)	<0·0001	7·4%	(6·2-8·7)	11·7%	(10·0-13·6)	24·3%	(21·7-27·1)	<0·0001
35-44	6·4%	(5·4-7·6)	5·7%	(4·8-6·8)	18·4%	(16·0-21·0)	<0·0001	5·9%	(4·9-7·2)	8·1%	(6·8-9·5)	12·2%	(9·9-14·9)	0·0025
**By number of sexual partners[2] in the past 5 years**														
0-1	8·8%	(7·9-9·7)	6·1%	(5·3-7·1)	21·8%	(20·0-23·7)	<0·0001	4·0%	(3·3-4·8)	5·3%	(4·3-6·6)	9·1%	(7·5-11·0)	0·0001
2-4	13·0%	(11·3-15·0)	10·7%	(9·2-12·3)	30·3%	(27·7-33·0)	<0·0001	6·1%	(4·9-7·5)	9·4%	(7·7-11·4)	17·0%	(14·4-19·8)	<0·0001
5-9	17·7%	(13·7-22·6)	14·4%	(11·2-18·3)	44·8%	(40·1-49·6)	<0·0001	12·3%	(9·3-16·0)	14·0%	(11·1-17·5)	26·4%	(22·2-31·0)	<0·0001
10+	25·1%	(15·6-37·9)	21·4%	(16·1-28·0)	49·2%	(42·6-55·8)	<0·0001	18·5%	(14·4-23·5)	20·1%	(16·6-24·1)	41·6%	(36·4-47·0)	<0·0001
**Denominators[3,4,5]**	6533, 5635		5603, 4944		4967, 3458			5088, 5837		4113, 4789		3429, 3502		
**Among target groups[6]**														
People who attended a GUM or sexual health clinic in the past 5 years	30·4%	(23·3-38·6)	36·3%	(31·1-41·8)	58·3%	(55·3-61·4)	<0·0001	38·0%	(30·8-45·7)	45·8%	(40·0-51·7)	50·8%	(46·4-55·1)	0·1819
Those diagnosed with an STI in the past 5 years[7]			29·9%	(22·4-38·7)	68·3%	(62·4-73·6)	<0·0001			31·0%	(22·7-40·8)	54·7%	(47·1-62·1)	0·0002
Women who attended antenatal services past 5 years			14·8%	(13·0-16·7)	47·9%	(44·7-51·0)	<0·0001							
Women who had an abortion in the past 5 years	18·5%	(14·5-23·3)	17·1%	(13·1-22·2)	48·8%	(42·5-55·2)	<0·0001							
Men who have had sex with a Man in the past 5 years								33·6%	(23·3-45·8)	39·3%	(31·5-47·7)	51·6%	(41·1-61·9)	0·0711
People of black African ethnic origin[8]			44·3%	(31·7- 57·7)	46·1%	(35·6-57·0)	0·8306			32·1%	(20·2-46·9)	43·9%	(30·3-58·6)	0·2429
People of any Black ethnic origin[8]	22·8%	(12·8-37·4)	30·3%	(24·1-37·2)	50·7%	(41·5-59·9)	0·0004	2·9%	(0·9-8·9)	22·5%	(16·2-30·4)	36·8%	(27·0-47·7)	0·0233
Population not in a target group[9]			3·5%	(2·9-4·2)	9·1%	(7·9-10·4)	<0·0001			5·8%	(5·0-6·7)	7·2%	(6·1-8·6)	0·0502

**Denominators[3,4] among target groups[6]**						
People who attended a GUM or sexual health clinic in the past 5 years	220, 185	452, 338	1270, 737	224, 247	372, 365	811, 676
Those diagnosed with an STI in the past 5 years[7]		168, 139	376, 210		120, 113	243, 214
Women who attended antenatal services, past 5 years		1768, 1547	1326, 872			
Women who had an abortion in the past 5 years		407, 343	326, 258			
Men who have had sex with a Man in the past 5 years				95, 98	155, 129	134, 112
People of black African ethnic origin[8]		88, 41	96, 75		67, 45	71, 82
People of any Black ethnic origin[8]	72, 65	236, 120	170, 130	66, 85	164, 128	117, 131
Population not in a target group[9]		3221, 2921	2377, 1803		3471, 3471	2601, 2389

Denominator is those aged 16-44 with 1+ partner, ever; unwt=unweighted, wt=weighted denominators.

[1] Question wording for sexual health clinic attendance was changed for Natsal-3 to reflect changes in services available at the time: participants in Natsal-1 and -2 were asked *"Have you ever attended a sexually transmitted disease (STD) clinic or special (VD) clinic?"*; participants in Natsal-3 were asked *"Have you ever attended a sexual health clinic (GUM clinic)?"*.

[2] Includes both opposite-sex and same-sex partners

[3] Unweighted, weighted denominators

[4] Denominator is participants aged 16-44 with 1+ partner, ever

[5] Denominators may vary across variables due to item non-response

[6] Target groups identified in UK National Guidelines for HIV Testing 2008. Abortion in past 5 years used as a proxy for attending a Termination of Pregnancy Clinic.

[7] Diagnosed with Chlamydia, Gonorrhoea, Herpes, Genital Warts, Trichomonas, NSU/NGU, Syphilis

[8] Full breakdown of ethnicity not available for Natsal-1, therefore in this table we present both 'Black African' ethnicity and 'Any Black ethnicity', to enable comparisons.

[9] Only derived for Natsal-2 and -3, because data not available for all target groups for Natsal-1. Includes those of 'Any Black Ethnicity' who are not of 'Black African ethnicity'.

[10] p-values for logistic regression comparing Natsal-3 with Natsal-2
